# Statistically Validated Networks in Bipartite Complex Systems

**DOI:** 10.1371/journal.pone.0017994

**Published:** 2011-03-31

**Authors:** Michele Tumminello, Salvatore Miccichè, Fabrizio Lillo, Jyrki Piilo, Rosario N. Mantegna

**Affiliations:** 1 Department of Social and Decision Sciences, Carnegie Mellon University, Pittsburgh, Pennsylvania, United States of America; 2 Dipartimento di Fisica, Università di Palermo, Palermo, Italy; 3 Santa Fe Institute, Santa Fe, New Mexico, United States of America; 4 Scuola Normale Superiore di Pisa, Pisa, Italy; 5 Department of Physics and Astronomy, Turku Centre for Quantum Physics, University of Turku, Turun yliopisto, Finland; Tel Aviv University, Israel

## Abstract

Many complex systems present an intrinsic bipartite structure where elements of one set link to elements of the second set. In these complex systems, such as the system of actors and movies, elements of one set are qualitatively different than elements of the other set. The properties of these complex systems are typically investigated by constructing and analyzing a projected network on one of the two sets (for example the actor network or the movie network). Complex systems are often very heterogeneous in the number of relationships that the elements of one set establish with the elements of the other set, and this heterogeneity makes it very difficult to discriminate links of the projected network that are just reflecting system's heterogeneity from links relevant to unveil the properties of the system. Here we introduce an unsupervised method to statistically validate each link of a projected network against a null hypothesis that takes into account system heterogeneity. We apply the method to a biological, an economic and a social complex system. The method we propose is able to detect network structures which are very informative about the organization and specialization of the investigated systems, and identifies those relationships between elements of the projected network that cannot be explained simply by system heterogeneity. We also show that our method applies to bipartite systems in which different relationships might have different qualitative nature, generating statistically validated networks in which such difference is preserved.

## Introduction

In recent years, many complex systems have been described and modeled in terms of bipartite networks [1]–[5]. Examples include movies and actors [1], [2], [4], authors and scientific papers [6]–[9], email accounts and emails [10], mobile phones and phone calls [11], plants and animals that pollinate them [12], [13]. One ubiquitous property of bipartite complex systems is their heterogeneity. For example, in a given period of time, some actors play in many movies, whereas others play in a few, some authors write a few papers, whereas others write many. Movies are also heterogeneous because of the size of cast, as well as papers because of the number of authors. Heterogeneity is also a common feature of biological complex systems. The genome of some organisms might contain a small set of proteins performing a given class of biological functions whereas the corresponding set of proteins is large for other organisms. Bipartite networks are composed by two different sets of nodes such that every link connects a node of the first set with a node of the second set. The properties of bipartite complex systems are often investigated by considering the one-mode projection of the bipartite network. One creates a network of nodes belonging to one of the two sets and two nodes are connected when they have at least one common neighboring node of the other set. In this paper we deal with the problem of identifying preferential links in the projected network. Specifically we use the term *preferential link* to indicate a link whose presence in the projected network cannot be explained in terms of random co-occurrence of neighbors in the bipartite system. We argue that these preferential links carry relevant information about the structure and organization of the system. When one constructs a projected network with nodes from only one set, the system heterogeneity makes it very difficult to discriminate preferential links from links which are consistent with a random null hypothesis taking into account the heterogeneity of the system. It is therefore of great importance to devise a method allowing to statistically validate whether a given link in the projected network is consistent or not with a null hypothesis of random connectivity between elements of the bipartite network.

The paper is organized as follows. In the Section Methods, we introduce our method to obtain a statistically validated network. In the Section Results and Discussion we first consider a *network of organisms*. Specifically, we obtain and discuss the statistically validated network of organisms used to define the clusters of orthologous genes database. We then study the *network of stocks* of the system of 500 stocks traded in the US equity markets and we point out that the statistically validated network of this section presents links describing a set of different relationships among the elements of the considered complex system. The last set of results concerns the *network of movies* where we consider the social bipartite system of movies and actors and we obtain statistically validated networks of movies. These networks are investigated with respect to their community structure and community characterization in the Text S1, where a few illustrative case studies of the informativeness of movies communities detected in statistically validated networks are provided. Finally, we draw some conclusions.

## Methods

**Figure 1:**
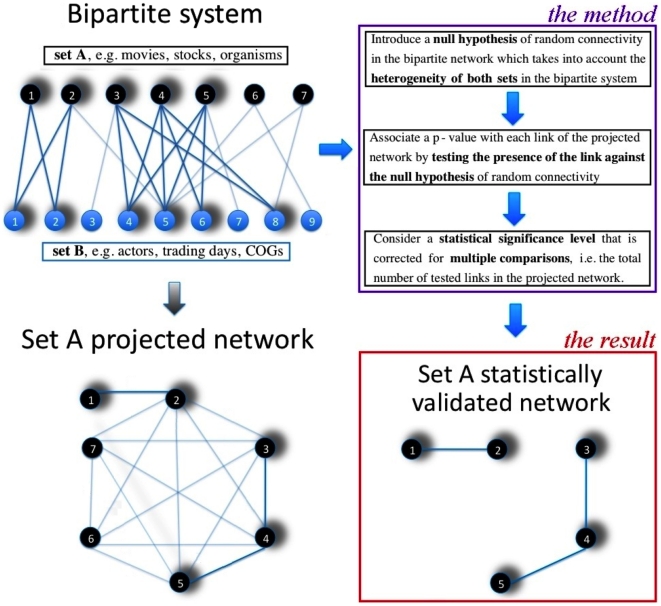
Illustrative example of the method. Illustrative example describing the method introduced to construct statistically validated networks in bipartite complex system.

Here we introduce an unsupervised method to statistically validate each link of the projected network. A schematic summary of our method is provided in Fig. 1. The key ingredients of our method are (i) the selection of a null hypothesis of random connectivity between elements in the bipartite network consistent with the degree of heterogeneity of both sets of elements, (ii) the identification of an analytical or computationally feasible procedure to associate a 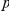-value with each link of the projected network, in order to test the presence of the link against the selected null hypothesis, and (iii) the appropriate correction of the statistical significance level in the presence of multiple hypothesis testing [14], [15] of links across the network.

### Statistically validated networks

**Figure 2:**
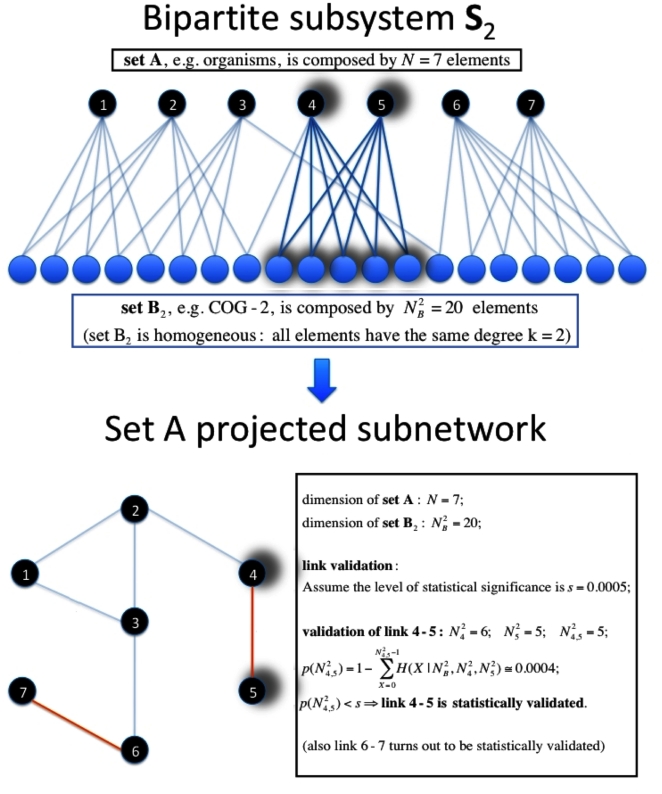
Illustrative example of the link validation procedure. Illustrative example describing the procedure introduced to validate the link between node 4 and 5 in the projected network of set A associated with the subsystem S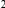 of a bipartite complex system. From the bipartite subsystem we note that the degree of elements 4 and 5 is 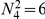 and 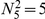 respectively. The number of elements of set B common to this pair of elements is 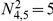. The computation of the p-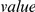 and his comparison with the chosen multiple hypothesis testing correction (s = 0.0005 in the example) is given in the box of the figure. For the illustrated subsystem and for the chosen multiple hypothesis testing correction the link 6–7 is also statistically validated.

The method works as follows. Let us consider a bipartite system **S** in which links connect the 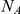 elements of set A to the 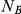 elements of set B. In the present discussion, we focus on the projected network on set A but the same approach is also valid when considering the projected network on set B. The adjacency projected network is obtained by linking together those vertices of A which share at least a common first neighbor element of B in the bipartite system. We aim to statistically validate each link of the projected network against a null hypothesis of random co-occurrence of common neighbors that takes into account the degree heterogeneity of elements of both set A and set B. In order to accomplish this goal we first decompose the bipartite system in subsystems. Fig. 2 shows an illustration of the link validation procedure in a specific subsystem. Each subsystem **S** 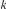 consists of all the 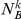 elements of set B with a given degree 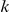 and of all the elements from set 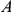 linked to them. By construction, a subsystem **S** 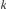 is homogeneous with respect to the degree of elements belonging to set B, because they all have the same degree 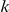. We indicate the set of elements of B with a certain degree 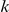 as set B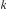. In the bipartite subsystem **S** 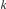 we are therefore left just with heterogeneity of elements of set A. Let us consider now two elements 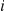 and 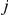 of set A, and assume they have 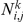 common neighbors in set B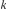. We denote the degree of elements 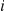 and 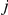 in the subsystem **S** 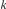 as 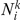 and 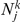, respectively. Under the hypothesis that elements 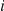 and 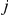 randomly connect to the elements of set B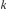, the probability that elements 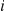 and 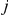 share 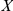 neighbors in set B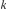 is given by the hypergeometric distribution [16], i.e.
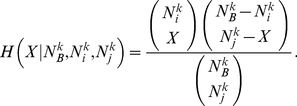
(1)


It is worth to mention that this distribution is symmetric with respect to exchange of elements 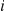 and 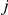, i.e. 
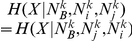
. The distribution given in Eq. (1) allows one to associate a p-value 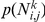 with the actual number 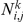 of neighbors that elements 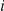 and 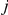 share:
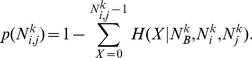
(2)


This way we have shown how to associate a p-value with the link between each pair of elements 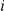 and 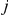 of the projected network for each subsystem **S** 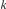. The next step of the method is to set a level of statistical significance 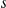, which takes into account the fact that we are performing multiple hypothesis testing - specifically a test for each pair of elements of 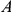 for each subsystem **S** 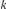. If we consider that the degree of elements of set B in the bipartite system ranges between 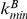 and 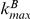 then the total number of tests that we perform will be 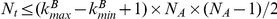. In the following examples, we will use a statistical level of significance of 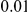 corrected for the 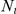 multiple comparisons in two different ways. Specifically we will use the very conservative Bonferroni correction [14], i.e. 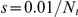 for multiple hypothesis testing and the less restrictive False Discovery Rate (FDR) [15]. For the moment, let us just assume that a value of statistical significance 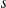 has been set, and proceed in the construction of the statistically validated network. We compare each p-value 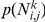 with 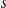. If 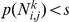 then we validate the link between elements 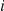 and 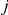 for the specific subsystem **S** 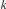. We then summarize all validations obtained in the projected adjacency network and associate with the link between 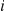 and 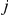 a weight equal to the total number of subsystems **S** 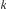
s in which the relationship between 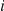 and 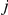 has been statistically validated. If the weight of a link turns out to be zero then the link is removed. The resulting weighted network is the aimed statistically validated network. Of course the obtained statistically validated network depends on the way we set the statistical threshold 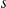. We name the statistically validated network obtained by setting 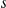 according to the Bonferroni correction as *Bonferroni network*. A less stringent correction for multiple hypothesis testing is the False Discovery Rate (FDR) [15]. The FDR correction for multiple hypothesis testing is defined as follows. Specifically, 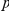-values of different tests are first arranged in increasing order (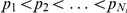), and the FDR threshold is obtained by finding the largest 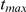 such that 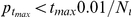. It is worth noting that by construction, the Bonferroni network is always a subnetwork of the FDR network. The advantage of using the FDR network is the fact that it allows one to include more interactions in the network, because the FDR correction is less restrictive than the Bonferroni correction. On the other hand, interactions included in the Bonferroni network are on average statistically more robust than interactions included in the FDR network. In this paper, we also consider the FDR correction and we refer to the network obtained by using it as the *FDR network*.

We apply our method to three different systems, namely the set of clusters of orthologous genes (COG) detected in completely sequenced genomes [17], [18], a set of daily returns of 500 US financial stocks, and the set of world movies of the IMDb database (http://www.imdb.com/). In the first set of COGs we can fully take into account both sources of heterogeneity of COGs and organisms. In the second set of excess returns of 500 US financial stocks the second source of heterogeneity is quite limited and therefore it is neglected. The last example presents a very large system with a high degree of heterogeneity of set B (actors) that cannot be efficiently taken into account with our method. However the second source of heterogeneity, although very large in absolute terms it is quite limited in relative terms with respect to the full size of the system. For this reason, although the statistically validated networks we obtain by neglecting the second source of heterogeneity are approximated, we show that they are fully informative about this large heterogeneous complex system. Moreover, we also show that the role of actors heterogeneity can be heuristically taken into account in the analysis of movies communities detected in the statistically validated networks. We choose to analyze these three systems because they are of interest in three different areas of science and they are different in size and level of heterogeneity, giving us the opportunity to show the power of our method under quite different conditions.

## Results and Discussion

### Network of organisms

The COG database [17], [18] provides the relationship between organisms and clusters of orthologous proteins present in their genome. Orthologous proteins have evolved from an ancestral protein and are likely to perform similar biological tasks in different genomes. By monitoring COGs across organisms one can therefore track the presence of different proteins involved in similar biological processes in different organisms. A projected network of organisms based on the co-occurrence of specific COGs might therefore highlight the degree of similarity of two organisms based on the functional characteristics of proteins present in their genome. Set A of the database is composed by 66 organisms (13 Archaea, 50 Bacteria and 3 unicellular Eukaryota) and set B by 4,873 COGs present in their genomes. The number of COGs in a genome is heterogeneous, ranging from 362 to 2,243. Similarly, COGs can be present in a different number of genomes. We call any COG that is present in 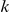 different genomes a 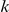-COG. In the present system, 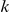 ranges between 3 and 66. We consider the projected network of organisms, in which we set a link between two organisms if at least one COG is present in the genome of both organisms. In the following we will refer to this network as the adjacency network of organisms, which turns out to be a complete network. The statistically validated networks are obtained by performing the procedure described in the previous Section. First we divide the bipartite system into COG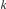 subsystems. Each COG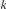 (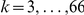) bipartite subsystem is characterized by the fact that all the COGs involved in it are 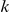-COGs. In each COG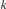 subsystem we are therefore left only with the heterogeneity of organisms. We test the existence of a preferential relationship between each pair of organisms separately for each COG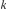 subsystem. Specifically, given two organisms 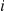 and 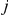, let 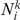 be the number of 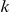-COGs in organism 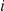, 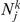 the number of 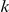-COGs in organism 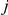 and 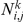 the number of 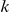-COGs belonging to both 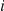 and 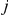. Under the null hypothesis of random co-occurrence, the probability of observing 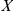 co-occurrences is given by 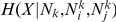 where 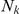 is the total number of 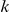-COGs in the system. We can therefore associate a 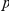-value to the observed 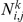 as described in Eq. 2. The described link validation procedure involves multiple hypothesis testing and therefore the statistical threshold must be corrected for multiple hypothesis testing. In our case the number of organisms is 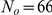 and we test 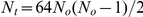 hypotheses, equal to the number of pairs of organisms times the number of COG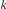 subsystems. Thus our Bonferroni threshold is 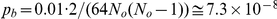. Each validated link has a weight equal to the total number of subsystems COG
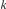
s in which the relationship between 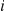 and 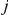 has been statistically validated.

**Figure 3:**
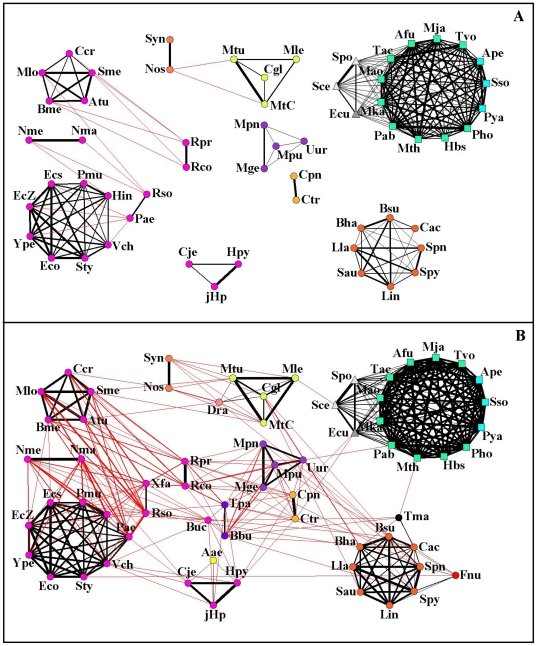
Statistically validated networks of organisms. Bonferroni (Panel A) and FDR (Panel B) networks of the organisms investigated in the COG database. The shape of the node indicates the super kingdom of the organism: Archaea (squares), Bacteria (circles), and Eukaryota (triangles). The color of the node indicates the phylum of the organism. The thickness of the link is related to its weight and it is proportional to the logarithm of the number of COG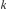 validations between the two connected nodes. Red links bridge different communities of organisms, as revealed by applying Infomap [22] to the statistically validated networks.

Let us now analyze the statistically validated networks obtained for this biological system. The Bonferroni network of organisms includes 58 non isolated nodes connected by 216 weighted links (Fig. 3A) and it shows seven connected components, each one having a clear biological interpretation in terms of organisms' lineage. The FDR network of organisms includes all the 66 organisms and the number of weighted links in this network is 369 (Fig. 3B). Thus the entire set is covered and the additional preferential links provide relations among the groups already observed in the Bonferroni network. The Bonferroni network (Fig. 3A) presents 7 connected components and 8 isolated nodes (isolated nodes are not shown in the figure). The largest connected component of the network, which is on the left in Fig. 3A, is composed by bacteria belonging to the phylum of Proteobacteria. Subgroups belonging to different classes can also be recognized. In fact, Eco, Ecz, Ecs, Ype, Hin, Pmu, Vch, Pae and Sty belong to the class of Gammaproteobacteria, whereas Atu, Sme, Bme, Ccr, Rpr, Rco and Mlo are Alphaproteobacteria and NmA, Nme and Rso are Betaproteobacteria. The second connected component is composed by Archaea genomes belonging to the two phyla of Euryarchaeota (Mth, Mja, Hbs, Tac, Tvo, Pho, Pab, Afu, Mka, and Mac) and Crenarchaeota (Pya, Sso and Ape). Archaea are also linked to the three unicellular eukaryotes present in the set, namely Ecu, Sce and Spo, although the weight of links between eukariotes and Archaea is markedly smaller than the weight of links among Archaea genomes [19]. The FDR network (Fig. 3B) is connected. However the group including Archaea and Eukaryota is clearly distinct from the network region of Bacteria. It is worth noting that both the Bonferroni and the FDR network display a clear clustered structure. Indeed the application of community detection algorithms [20], [21], such as Infomap [22], to the statistically validated networks reveal clusters of organisms with a direct biological interpretation in terms of lineage (see Fig. 3). This is not true for the adjacency network, and shows that the statistically validated networks are able to identify the many preferential links inside communities and the few preferential links bridging different communities of organisms.

### Network of financial stocks

As a second example we consider the collective dynamics of the daily returns of 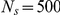 highly capitalized US financial stocks in the period 2001–2003 (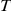 = 748 trading days). Many studies investigating correlation based networks have shown that the information about the different economic sectors of the quoted companies is incorporated into their price dynamics [23]. In this case, the two sets of the bipartite system are the stocks (with categorical information on their returns) and the trading days. Here we focus on the projected network of stocks. The interest in this example is that we (i) generalize our procedure to complex systems where the elements are monitored by continuous variables, (ii) show how to simplify the above procedure when the second source of heterogeneity (in the previous example the COG frequency in different organisms) is small, and (iii) show how to classify links according to the type of relation between the two nodes.

**Figure 4:**
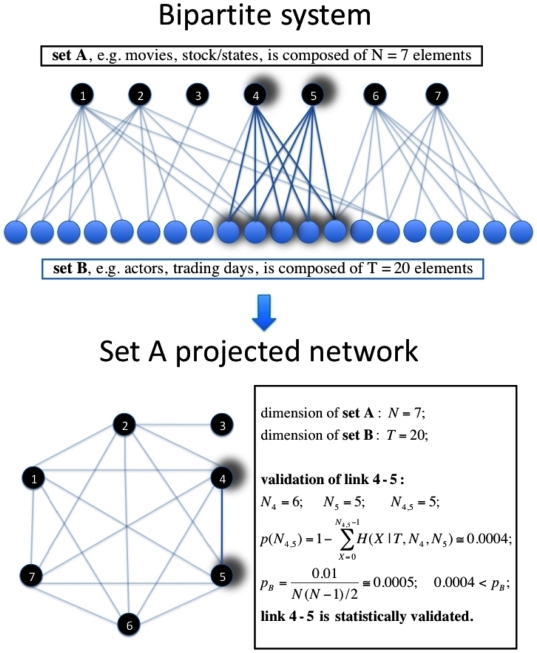
Illustrative example of the link validation procedure. Illustrative example describing the procedure introduced to validate a link in the projected network when the degree heterogeneity of Set B is negligible or cannot properly be taken into account. The example explicitly worked out in the box of the figure considers the validation of the link 4–5 of the projected network of set A. For these nodes the degree of elements 4 and 5 is 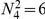 and 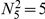 respectively. The number of elements of set B common to this pair of elements is 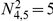. The computation of the p-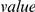 and his comparison with the Bonferroni multiple hypothesis testing correction (s = 0.0005 in the example) is given in the box of the figure.

Since we want to identify similarities and differences among stock returns not due to the global market behavior, we investigate the excess return of each stock 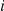 with respect to the average daily return of all the stocks in our set. The excess return of each stock 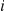 at day 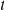 is then converted into a categorical variable with 3 states: *up*, *down*, and *null*. For each stock we introduce a daily varying threshold 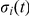 as the average of the absolute excess return (a proxy of volatility) of stock 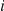 over the previous 20 days. State *up* (*down*) is assigned when the excess return of stock 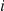 at day 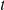 is larger (smaller) than 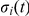 (-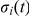). The state *null* is assigned to the remaining days. We study the co-occurrence of states *up* and *down* for each pair of stocks. In this case we can neglect the heterogeneity of state occurrence in different trading days because the number of *up* (*down*) states is only moderately fluctuating across different days and it has a bell shaped distribution with a range of fluctuations smaller than one decade for each stock. With this approximation we can statistically validate the co-occurrence of state 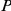 (either *up* or *down*) of stock 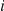 and state 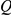 (either *up* or *down*) of stock 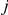 with the following procedure (illustrated in Fig. 4). Let us call 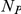 (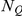) the number of days in which stock 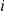 (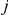) is in the state 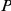 (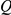). Let us call 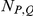 the number of days when we observe the co-occurrence of state 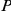 for stock 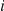 and state 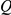 for stock 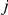. Under the null hypothesis of random co-occurrence of state 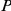 for stock 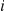 and state 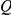 for stock 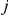, the probability of observing 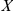 co-occurrences of the investigated states of the two stocks in 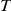 observations is again described by the hypergeometric distribution, 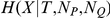. As before we can associate a 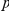-value with each pair of stocks for each combination of the investigated states. We indicate the state *up* (*down*) of stock 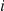 as 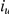 (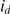). The possible combinations are (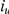, 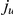), (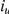, 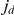), (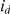, 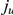), and (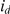, 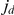). As before the statistical test is a multiple hypothesis test and therefore either the Bonferroni or FDR correction is necessary. The Bonferroni threshold is 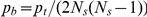 where the denominator of the threshold is the number of considered stock pairs (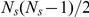) times 4, which is the number of different co-occurrences investigated. Each pair of stocks is characterized by the set of the above four combinations which are statistically validated. There are 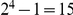 possible cases with at least one co-occurrence validation, but we observe only 5 kinds of preferential links: L1 in which the co-occurrences (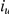, 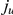) and (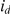, 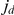) are both validated; L2 in which only the co-occurrence (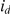, 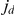) is validated, L3 in which only the co-occurrence (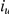, 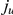) is validated, L4 in which either only (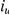, 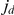) or only (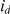, 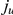) is validated; and L5 when both the co-occurrence (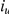, 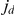) and (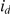, 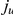) are validated. Note that we put in the same relationship L4 two cases which are different only for the order in which the two nodes are considered. The set of relationships 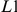, 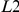, and 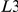 and the associated links describe a coherent movement of the price of the two stocks, while the set of relationships 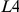 and 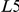 describes opposite deviation from the average market behavior. We can therefore construct networks where the statistically validated links are associated with a label that specifies the type of relationship between the two connected nodes. This structure is richer than a simple unweighted network, but it is also different from a weighted network because it describes relationships which cannot be described by a numerical value only. We address the set of different relationships present between two nodes of the statistically validated network with the term *multi-link*.

**Figure 5:**
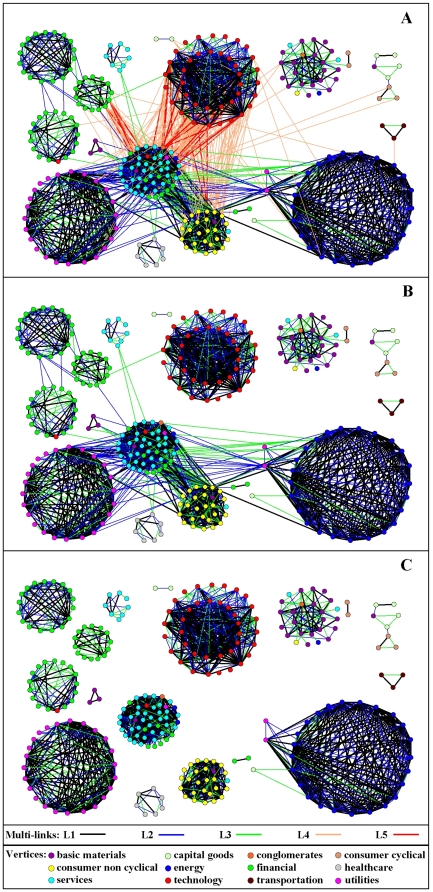
Bonferroni network of stocks. The largest connected component of the Bonferroni network associated with the system of 500 stocks. The nodes represent stocks and links connecting different stocks correspond to the statistically validated relationships. The node color identifies the economic sector of the corresponding stock. The economic sector classification is done according to Yahoo Finance. The color of a multi-link identifies the corresponding validated relationship. In panel A we report the largest connected component of the Bonferroni network. In panel B we remove links corresponding to anti-correlated evolution of stock returns, i.e. links 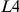 and 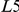. In panel C we also remove links bridging different clusters detected by the Infomap method.

The Bonferroni network of the system is composed by 349 stocks connected by 2,230 multi-links. The multi-links are of different nature. Specifically, we observe 1,158 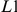-links, 494 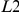-links, 354 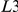-links, 196 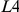-links, and 28 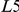-links. The largest connected component of the network includes 273 stocks. There are also 19 smaller connected components of size ranging from 2 to 15. In Fig. 5A we show the largest connected component of the Bonferroni network. It presents several regions in which stocks are strongly connected by 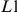, 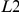, and 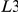 multi-links. These regions are very homogeneous with respect to the economic sector of the stocks. The connection between different regions is in some cases provided by a large number of 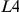 and 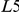 multi-links. This is especially evident for the group of technology stocks (red circles). All except one of the multi-links outgoing from the group are 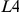 and 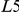 multi-links, indicating moderate or strong anti-correlation of technology stocks with the other groups. The strongest anti-correlation is detected between technology and services stocks (cyan circles).

The multi-link statistically validated network of 500 stocks is a new kind of network presenting qualitatively and quantitatively different classes of links. For this reason, there are no established methods specifically devised to detect communities of nodes in this kind of network. Here we propose a minimalist approach in which we just distinguish between co-occurrences of correlated evolution from co-occurrences of anti-correlated evolutions. Our procedure works as follows: first we remove all the links describing anti-correlated evolutions (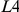 and 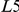) from the multi-link statistically validated network (see Fig. 5B). Then we weight the remaining links by taking into account whether the statistical validation of the link is single or twofold. With this choice, the twofold link 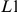 has a weight equal to 2, whereas single links 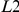 and 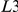 have a weight equal to 1. We then perform community detection on the resulting “standard” weighted network of Fig. 5B, by using the Infomap method [22]. While our approach is pragmatic and heuristic, we are aware that a more theoretically grounded approach to partitioning multi-link networks would certainly be useful in the study of networks where links of different nature can be naturally defined, as in the present case.

We analyze the clusters of stocks detected in the weighted Bonferroni network by using the information about the economic sectors and subsectors of stocks in each cluster. Economic sectors according to Yahoo Finance classification of stocks are Basic Materials, Capital Good, Conglomerates, Consumer Cyclical, Consumer Non Cyclical, Energy, Financial, Healthcare, Services, Technology, Transportation, Utilities. A statistical method to perform this analysis is given in Ref. [24]. The total number of economic sectors is 12, and they are detailed in Fig. 5. Economic subsectors represent a more detailed classification of stocks. There are 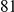 different subsectors characterizing the 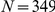 non isolated stocks in the Bonferroni network. The Infomap method detects 37 clusters of stocks with size ranging from 2 to 48 in the Bonferroni network. In Fig. 5C, we show the clusters of stocks obtained for the largest connected component of the Bonferroni network. It is evident from Fig. 5C that most of the clusters are very homogeneous in terms of the economic sector of stocks. However some clusters are better characterized in terms of subsectors. Let us for instance focus on the 3 clusters of financial stocks (green vertices in Fig. 5) at the top left corner in Fig. 5C. From top to bottom, these three clusters are composed by stocks belonging to the sub-sectors of insurance (life, and property and casualty), of investment services, and of regional banks. Another example is the cluster at the center of Fig. 5C, which is mostly composed by stocks of the services sector (cyan in the figure). These stocks belong to the sub-sector of services – real estate. It is to notice that this cluster is strongly anti-correlated (links L4 and L5 in Fig. 5A) with a large cluster of stocks belonging to the sector of technology (red vertices in Fig. 5).

We have also computed the FDR network of the system. As expected, it includes more stocks (494) and more multi-links (11,281) than the Bonferroni network, since the requirement on the statistical validation is less restrictive. The FDR network has a single connected component and the fraction of 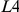 and 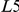 multi-links is higher (35.9%) than in the case of the Bonferroni network (10.0%).

As before the adjacency network of stocks is a complete graph. On the contrary both the Bonferroni and the FDR networks display a highly clustered structure with clusters having a clear economic meaning. The use of Infomap on these statistically validated networks gives a partition in communities, which are extremely homogeneous in terms of economic sector. Therefore our method allows to construct networks where (i) links are statistically validated, (ii) multi-links describe qualitatively different relationships between pairs of stocks, e.g. both co-movements and opposite movements occurring between pairs of stocks, and (iii) a very accurate identification of communities of stocks is possible. To the best of our knowledge the presence of all these features is pretty unique and it is not shared by other similarity networks [23] based on topological constraints [25]–[27], correlation threshold [28], [29], or validated with bootstrap [30].

### Network of movies

The last system we investigate is the bipartite system of movies and actors of the Internet Movie Database (IMDb), which is the largest web repository of world movies. We consider here the bipartite relationship between movies and actors produced in the period 1990–2008 all over the world. The set includes movies realized in 169 countries. We choose this system because (i) it is a large system (89,605 movies and 412,143 actors), (ii) it has a large heterogeneity both in movies and in actors, and (iii) it allows a sophisticated cluster characterization analysis based on the characteristics of the movie, namely genre, language, country, and filming locations.

The actors degree heterogeneity ranges between 1 and 247 and it is so pronounced that we did not find a practical solution to take it into account when constructing statistically validated networks of movies. The approach of the 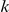-subsets is not feasible in this case due to lack of sufficient statistics. Therefore, we perform a statistical validation of links against a null hypothesis fully taking into account the movies heterogeneity but not describing the heterogeneity of actors. In spite of this limitation, the results obtained for the statistically validated networks are very informative about several aspects of the movie industry as it will be shown in the following. We conjecture that this is due to the fact that although the degree heterogeneity of actors is remarkable in absolute terms, making it unfeasible to use the 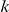-subset approach, it is small as compared with the total number of movies. Indeed the fraction between the maximum number of movies performed by a single actor in the database and the total number of movies is 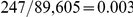. This fact indicates that no actors contribute systematically to increase the co-occurrence between all movies pairs, or even a relevant fraction of them. This situation is significantly different than the one observed for the system of organisms and COGs, where the maximum degree of COGs was 66, i.e. the same as the total number of organisms in the database.

We construct the statistically validated networks of movies by testing the co-occurrence of actors in the cast of each movie pair. A schematic representation of the procedure used to validate links is provided in Fig. 4. The null hypothesis of random co-coccurecence is again described by the hypergeometric distribution, which naturally takes into account the heterogeneity of the system due to the different size of the cast of movies. Table 1 shows the severe filtering of nodes and links that is obtained in the validated networks of movies with respect to the adjacency network. Only 16% (47%) of the nodes and 1% (7%) of the links of the adjacency network are statistically validated in the Bonferroni (FDR) network. Also the size of the largest connected component varies significantly across the three networks. Specifically the largest connected component (i) is covering almost completely the adjacency network, (ii) comprises the largest fraction of movies in the FDR network (83%), but (iii) contains only 13% of the movies of the Bonferroni network. This shows that the Bonferroni network already provides a natural partition of the movies included in it.

**Table 1:** Basic properties of movie networks.

	Movies	Links	Number of	Largest
			conn. comp.s	conn. comp.
Adjacency	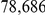	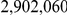	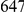	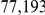
FDR	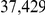	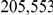	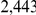	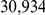
Bonferroni	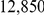	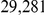	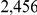	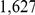

**Figure 6:**
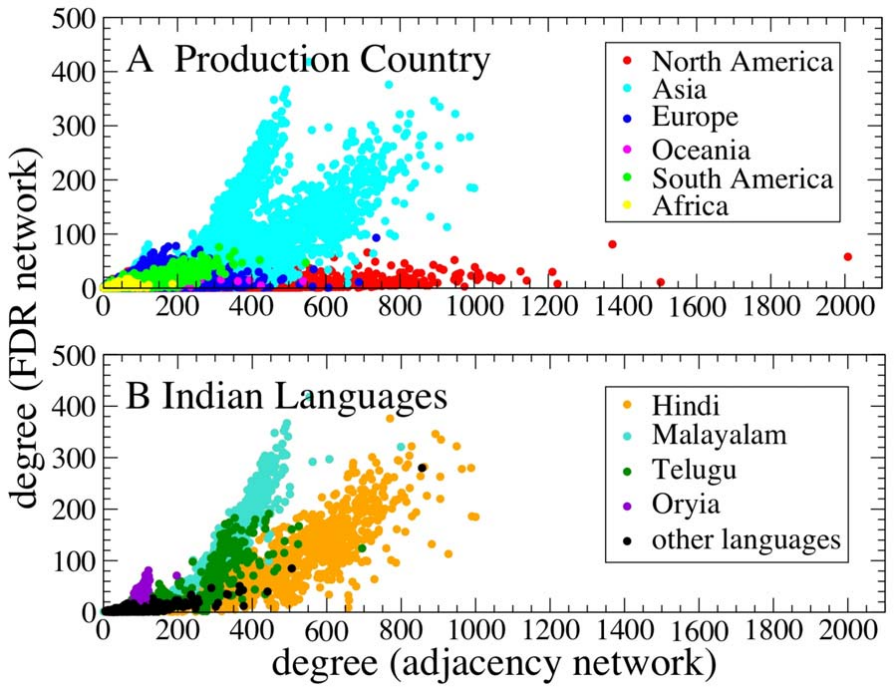
Comparison between adjacency and FDR networks of movies. Scatter plots of the degree of movies in the adjacency and FDR networks. Each circle represents a movie. We do not report movies with vanishing degree in at least one of the two networks. The panel A shows movies produced all over the world. The color of each symbol identifies the continent of the production country. Only movies with a single production country are shown. The panel B shows the data for the Indian movies and the color indicates the movie language. Only movies with a single language are shown.

A comparison of the degree of movies in the adjacency and FDR networks allows to clearly distinguish the Asian movie industry from the rest of the world movie industry, and different languages within single countries like India (see Fig. 6). The North American movie industry shows typically a high degree of movies in the adjacency network and a relatively low degree in the FDR network (see Fig. 6A), probably indicating a tendency to avoid a similar cast in different movies. A different behavior is observed in Asia, while Europe is an intermediate case. The analysis of indian movies (see Fig. 6B) shows the existence of groups of movies characterized by a common language. According to the present state of the IMDb database, the comparison between the degree of adjacency network and the degree of FDR network suggests that the Asian movie industry, and the Indian movie industry in particular, presents a level of variety in the cast formation that is lower than the variety observed in the western movie industry. In the Text S1, we analyze the movie communities detected when the Infomap method is applied to different movie networks. Specifically we investigate and compare the community structure of adjacency, FDR and Bonferroni networks. Different aspects of the comparison are summarized in Figure S1, and Tables S1, S2, and S3. In the community detection of adjacency and statistically validated networks we weight links according to Ref. [31] to heuristically take into account actors' heterogeneity in the number of performed movies. In the Text S1, we show that the clusters of movies obtained from the Bonferroni and FDR networks have a higher homogeneity in terms of production country, language, genre, and filming location than the clusters of movies detected from the adjacency network.

### Conclusions

In summary, our method allows to validate links describing preferential relationships among the heterogeneous elements of bipartite complex systems. Our method is very robust with respect to the presence of false positive links, i.e. links that might be just due to statistical fluctuations. In fact, we verified for all the investigated systems that the Bonferroni network associated with a random rewiring of the bipartite network turns out to be empty. By applying the method to three different systems, we showed that it is extremely flexible, since it can be applied to systems with different degree of heterogeneity and described by binary relationships and categorical variables.

## Supporting Information

Figure S1 **Rank plot of the size of clusters in the adjacency, Bonferroni and FDR networks.** Rank plot of the size of clusters obtained with the Infomap algorithm for the adjacency movie network, the FDR network and the Bonferroni network both for the unweighted and weighted links. The difference between the partitions decreases for the statistically validated networks (see text for a measure of the mutual information between unweighted and weighted partitions). In the legend, the number in parenthesis is the number of detected clusters in the corresponding network.
(TIFF)

Table S1 **Cluster over-expression analysis of production country, language, genre and filming location.** Clusters are obtained by performing the Infomap partitioning of the adjacency weighted movie network (ADJ-W), FDR weighted movie network (FDR-W) and the Bonferroni weighted movie network (BONF-W). For each of the four considered classifications, we report the total number of observed over-expressions for each network. The number in parenthesis is the number of distinct clusters where at least one over-expression has been observed.
(PDF)

Table S2 **Over-expression of production country (C), language (L), genre (G) and filming locations (F) for seven large clusters of the FDR weighted network.** Here we consider only those movies that are also present in cluster 1 of the adjacency weighted network (ADJ-W). In fact, the number in parenthesis indicates the number of movies in a specific FDR-W cluster that are also present in cluster 1 of the adjacency weighted movie network.
(PDF)

Table S3 **Over-expression of production country (C), language (L), genre (G) and filming locations (F) for two large clusters of FDR weighted network and five large clusters of Bonferroni weighted networks.** Here we consider the movies that are also present in cluster 24 of the adjacency weighted movie network. In fact, the number in parenthesis indicate the number of movies in a specific FDR-W or BONF-W cluster that are also present in cluster 24 of the adjacency weighted movie network.
(PDF)

Text S1 **Community detection and characterization.**
(PDF)
