# Supplementary figures and images for: Statistically Validated Networks in Bipartite Complex Systems
Source: PLoS One. 2011 Mar 31;6(3):e17994. doi: 10.1371/journal.pone.0017994 (PMC3069038; doi:10.1371/journal.pone.0017994)

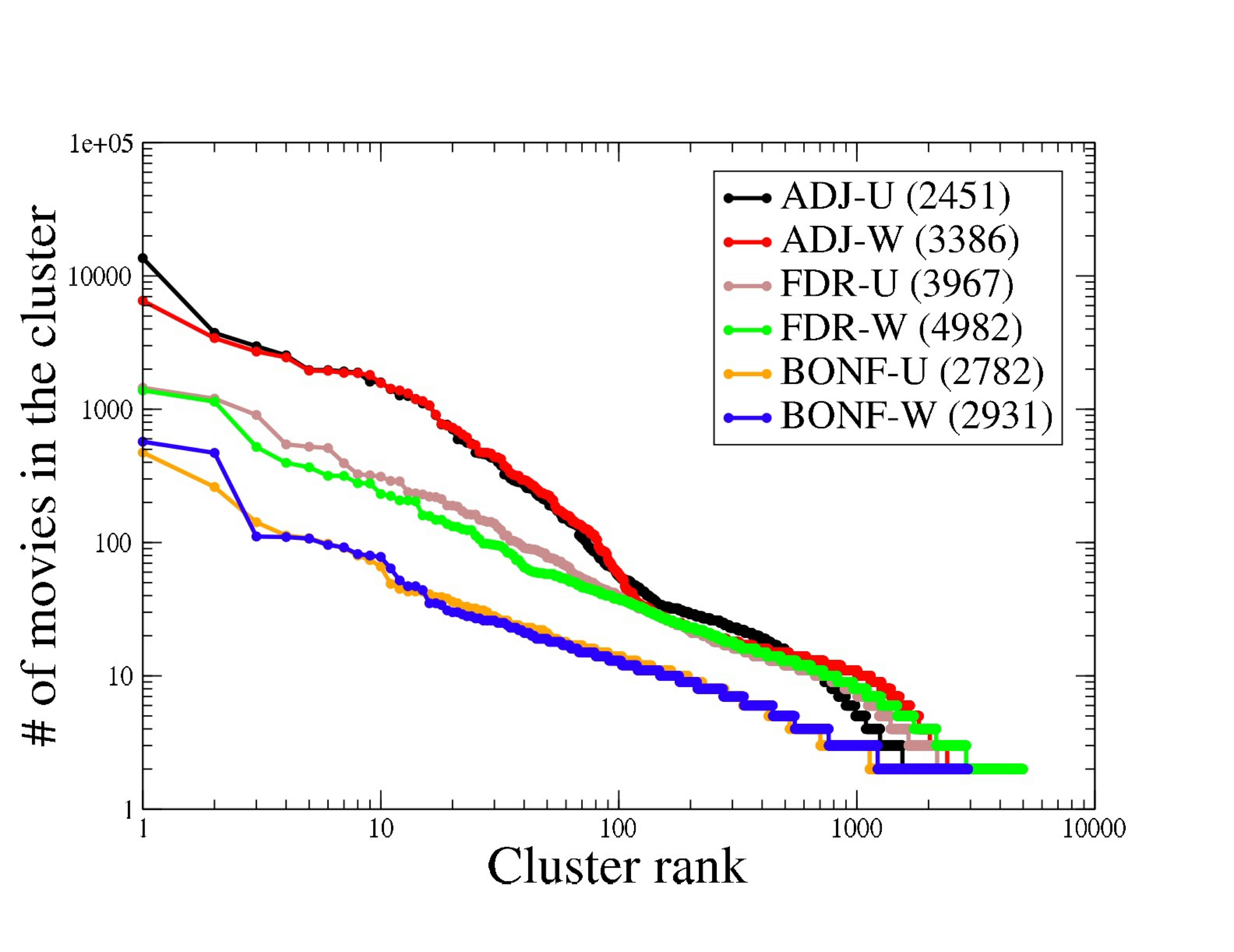

Supplement: Figure S1 — Rank plot of the size of clusters in the adjacency, Bonferroni and FDR networks. Rank plot of the size of clusters obtained with the Infomap algorithm for the adjacency movie network, the FDR network and the Bonferroni network both for the unweighted and weighted links. The difference between the partitions decreases for the statistically validated networks (see text for a measure of the mutual information between unweighted and weighted partitions). In the legend, the number in parenthesis is the number of detected clusters in the corresponding network. (TIFF) [file pone.0017994.s001.tif]
