# Supplementary material for: Statistically Validated Networks in Bipartite Complex Systems
Source: PLoS One. 2011 Mar 31;6(3):e17994. doi: 10.1371/journal.pone.0017994 (PMC3069038; doi:10.1371/journal.pone.0017994)
Supplement: Table S1 — Cluster over-expression analysis of production country, language, genre and filming location. Clusters are obtained by performing the Infomap partitioning of the adjacency weighted movie network (ADJ-W), FDR weighted movie network (FDR-W) and the Bonferroni weighted movie network (BONF-W). For each of the four considered classifications, we report the total number of observed over-expressions for each network. The number in parenthesis is the number of distinct clusters where at least one over-expression has been observed. (PDF) [file pone.0017994.s002.pdf]

Table S1: Cluster over-expression analysis of production country, language, genre and filming location. Clusters are obtained by performing the Infomap partitioning of the adjacency weighted movie network (ADJ-W), FDR weighted movie network (FDR-W) and the Bonferroni weighted movie network (BONF-W). For each of the four considered classifications, we report the total number of observed over-expressions for each network. The number in parenthesis is the number of distinct clusters where at least one over-expression has been observed.

	ADJ-W	FDR-W	BONF-W
movies in all clusters	78,686	37,429	12,850
number of clusters	3,386	4,982	2,931
Production country	1,206 (1,115)	1,944 (1,816)	1,009 (960)
Language	601 (494)	1,429 (1,297)	819 (729)
Genre	629 (445)	715 (533)	373 (281)
Filming location	2,196 (793)	1,836 (1,123)	853 (571)
