# Supplementary material for: Statistically Validated Networks in Bipartite Complex Systems
Source: PLoS One. 2011 Mar 31;6(3):e17994. doi: 10.1371/journal.pone.0017994 (PMC3069038; doi:10.1371/journal.pone.0017994)
Supplement: Table S3 — Over-expression of production country (C), language (L), genre (G) and filming locations (F) for two large clusters of FDR weighted network and five large clusters of Bonferroni weighted networks. Here we consider the movies that are also present in cluster 24 of the adjacency weighted movie network. In fact, the number in parenthesis indicate the number of movies in a specific FDR-W or BONF-W cluster that are also present in cluster 24 of the adjacency weighted movie network. (PDF) [file pone.0017994.s004.pdf]

Table S3 : Over-expression of production country (C), language (L), genre (G) and filming locations (F) for two large clusters of FDR weighted network and five large clusters of Bonferroni weighted networks. Here we consider the movies that are also present in cluster 24 of the adjacency weighted movie network. In fact, the number in parenthesis indicate the number of movies in a specific FDR-W or BONF-W cluster that are also present in cluster 24 of the adjacency weighted movie network.

cluster	FDR-W 5	FDR-W 43	BONF-W 10	BONF-W 13	BONF-W 309	BONF-W 607	BONF-W 806
movies	396 (390)	132 (125)	111 (111)	110 (110)	13 (13)	10 (10)	15 (13)
C	India 395	India 132	India 111	India 110	India 13	India 10	India 15
L	Telugu 375	Tamil 120	Telugu 111	Telugu 109	Telugu 12	Telugu 9	Tamil 14
L	Tamil 15	Hindi 21	-	-	-	-	-
L	-	Telugu 21		-	-	-	-
G	Action 132	Romance 52	Family 25	Action 47	-	-	-
G	Romance 94	Action 48	Musical 11	Romance 39	-	-	-
G	Family 40	Musical 17	-	-	-	-	-
G	Musical 24	-	-	-	-	-	-
F	NA 315	NA 99	NA 98	NA 78	-	-	-
F	Hyderabad 45	Chennai 15	-	Hyderabad 13	-	-	-
F	Andhra Pradesh 7	Tamil Nadu 3	-	Andhra Pradesh 5	-	-	-
F	Rajahmundry 4	-	-	-	-	-	-
