# Supplementary material for: Statistically Validated Networks in Bipartite Complex Systems
Source: PLoS One. 2011 Mar 31;6(3):e17994. doi: 10.1371/journal.pone.0017994 (PMC3069038; doi:10.1371/journal.pone.0017994)
Supplement: Table S2 — Over-expression of production country (C), language (L), genre (G) and filming locations (F) for seven large clusters of the FDR weighted network. Here we consider only those movies that are also present in cluster 1 of the adjacency weighted network (ADJ-W). In fact, the number in parenthesis indicates the number of movies in a specific FDR-W cluster that are also present in cluster 1 of the adjacency weighted movie network. (PDF) [file pone.0017994.s003.pdf]

**Table S2 :** Over-expression of production country (C), language (L), genre (G) and filming locations (F) for seven large clusters of the FDR weighted network. Here we consider only those movies that are also present in cluster 1 of the adjacency weighted network (ADJ-W). In fact, the number in parenthesis indicates the number of movies in a specific FDR-W cluster that are also present in cluster 1 of the adjacency weighted movie network.

| cluster | 17 | 56 | 188 | 47 | 91 | 123 | 95 |
|---|---|---|---|---|---|---|---|
| movies | 98 (87) | 59 (58) | 48 (46) | 46 (41) | 53 (39) | 45 (37) | 45 (33) |
| C | USA 88 | USA 58 | USA 47 | USA 44 | USA 53 | USA 45 | USA 44 |
| L | English 93 | English 57 | English 46 | English 46 | English 49 | English 43 | English 45 |
| G | Animation 77 | Action 47 | Horror 12 | Comedy 38 | Comedy 33 | Comedy 34 | Thriller 18 |
| G | Family 62 | - | Sci-Fi 12 | - | Music 6 | - | Crime 14 |
| G | Adventure 45 | - | - | - | - | - | |
| G | Fantasy 29 | - | - | - | - | - | |
| G | Sci-Fi 15 | - | - | - | - | - | |
| G | Musical 9 | - | - | - | - | - | |
| F | NA 75 | Los Angeles 19 | - | Los Angeles 30 | Los Angeles 21 | Los Angeles 25 | Miami 23 |
| F | - | - | - | Long Beach 5 | - | - | Miami Beach 9 |
| F | - | - | - | - | - | - | Fort Lauderdale FL 6 |
| F | - | - | - | - | - | - | Florida 5 |
| F | - | - | - | - | - | - | Coral Gables FL 4 |
| F | - | - | - | - | - | - | Hollywood FL 3 |
| F | - | - | - | - | - | - | Key Biscayne FL 3 |
