# Supplementary material for: Statistically Validated Networks in Bipartite Complex Systems
Source: PLoS One. 2011 Mar 31;6(3):e17994. doi: 10.1371/journal.pone.0017994 (PMC3069038; doi:10.1371/journal.pone.0017994)
Supplement: Text S1 — Community detection and characterization. (PDF) [file pone.0017994.s005.pdf]

Community detection and characterization

Community detection in the movie networks and its characterization

We perform community detection [1, 2] on the adjacency and statistically validated networks, in order to put in evidence the different community structure of these networks. We obtain a partition of the vertices of networks by using the Infomap method by Rosvall and Bergstrom [3]. This algorithm allows to efficiently investigate both weighted and unweighted networks. For each investigated network, we run the Infomap 10^3 times and we select the best partition according to the minimal "code length" [3]. The obtained partition depends on whether the network is weighted or not, and eventually on how weights are selected. Therefore, in the following we discuss case by case the way in which cluster detection is performed. Once clusters of elements are detected, there still remains the problem of cluster interpretation. We address this problem by comparing the partition of the system as produced by the Infomap with an *a priori* classification of the elements of the system. For instance movies can be characterized by their genre, and stocks can be characterized according to their economic sector.

The largest component of the adjacency movie network comprises 77,193 movies whereas the second largest component has only 11 movies. When we apply the Infomap partitioning algorithm to the unweighted adjacency movie network we obtain a partitioning of the network which presents 2,451 distinct clusters. The cluster size decreases smoothly from the largest value of 13,608 down to the smallest value of 2. We will see in the following discussion that the partitioning of the network presents a certain degree of informativeness about the system. In fact the obtained clusters present a certain degree of homogeneity with respect to the main country of production, the language and some classes of genre of movies. The FDR network is characterized by a largest connected component of 30,934 movies. The Infomap algorithm makes a partition of this and other components of the network into 3,967 clusters whose size is decreasing from 1,478 to 2 movies. Table 1 of the main paper shows that the Bonferroni network does not present a giant connected component. In fact the largest connected component comprises only 13% of the movies linked in the network. However, the application of the Infomap algorithm refines the natural partitioning of the network by detecting 2,782 clusters whose size is ranging from 577 to 2 movies. We also note that the number of connected components in the Bonferroni network (2,456) is roughly equal to the number of the Infomap clusters in the adjacency network (2,451).

Our method provides a full control of the statistical validation of links against a random null hypothesis

taking into account the fact that different movies have a different number of actors in their cast. We have already discussed that the system also presents a second source of heterogeneity. In fact, different actors typically play a different number of movies. We do not have a rigorous and computationally feasible way to also take into account this second source of heterogeneity in our statistical validation procedure. We therefore use a heuristic approach, and take into account this heterogeneity by following M. E. J. Newman. Specifically, we adapt the procedure proposed in the paper [4] to our system by weighting each link of the projected networks as follows. We associate a weight w_{ab} with each pair of linked movies a and b such that the weight takes into account the different number of movies played by the actors playing both the movies a and b. Specifically,

$$w_{ab} = \sum_{i=1}^{Q} \frac{1}{N_i - 1}, \tag{1}$$

where the sum is taken over all the Q actors who played both movie a and b, and N_i is the total number of movies played by actor i. By performing community detection on the weighted adjacency network of movies, we obtain a more refined partitioning of the 78,686 movies present in the network. Specifically, the clusters obtained with the Infomap algorithm present 3,386 clusters whose size is decreasing from 6,523 to 2. By performing community detection on the weighted statistically validated networks, we obtain results that are very similar to those obtained for the corresponding unweighted networks. The impact of considering link weights in community detection is quantitatively discussed in the following subsection.

The Infomap method allows to take into account link weights. This feature implies that results of community detection in a given network may significantly change when weights of links are considered. For each network, we quantify the difference between the partition obtained without using link weights and the partition obtained by taking link weights into account by calculating the (normalized) mutual information between the two partitions [5]. The mutual information takes the maximum value of 1 for two identical partitions of the network. We observe a value of 0.798, 0.913 and 0.976 for the adjacency, FDR and Bonferroni networks, respectively. We therefore observe a net increase of the mutual information when we consider statistically validated networks. Furthermore, the mutual information reaches a value very close to 1 for the Bonferroni network, which is obtained under the most restrictive statistical requirements. In other words, we observe that the source of heterogeneity of the actors' productivity has a minor impact into the partitioning of the statistically validated networks, especially for the Bonferroni network.

In the following, we separately discuss the results obtained for the partitioning of the adjacency, FDR and Bonferroni weighted networks. Results obtained for the Bonferroni network are rather similar to those obtained for the FDR network. The size profile of the clusters obtained by partitioning the adjacency, FDR and Bonferroni networks are shown in Fig. S1, both in the case of unweighted and weighted networks. It is quite clear from the figure that the cluster size decreases from the largest to the smallest cluster in a pretty different way for the adjacency networks and the statistically validated networks. In fact, the FDR and Bonferroni networks present a decay of cluster size versus its rank that is well approximated by a power-law decay.

In the majority of cases, the clusters detected in the Bonferroni network correspond to the strongest interconnected parts of larger clusters detected in the FDR network. The clusters of the FDR network correspond in turn to sets of movies which in large majority are present in bigger clusters observed in the weighted adjacency network. This general observation should not be seen as a strict inclusive relation but we wish to point out that a sort of "typical" inclusiveness is observed for most of the detected clusters.

Community characterization.

We analyze the clusters of movies we obtain for the three different weighted networks, by considering the over-expression of specific characteristics of the movies contained in each cluster. Specifically, we consider 4 different classifications of movies obtained by using the information about movies stored into the IMDb. Indeed the IMDb reports for each movie indication about (i) country or countries of production, (ii) language or languages used in the movie, (iii) movie genre (or genres) and (iv) location or locations where the movie was shot. Only in a limited number of cases some of these classifications are not available. When this happens we indicate the missing attribute about the movie as "not available". We characterize clusters obtained for all the networks by separately testing the over-expression of each attribute present in each one of the above mentioned 4 classifications. The method used to quantitatively characterize the clusters is described in Ref. [6].

In different networks we observe a different profile of over-expression. The degree of specificity is higher for smaller clusters and therefore a higher specificity is observed for the Bonferroni and for the FDR networks. This is especially true for the genre and the location classifications. In general, the country and language over-expression is quite specific for most clusters in the investigated networks. Exceptions are clusters containing movies produced in former Yugoslavia and Soviet Union. This is

due to the fact that during the investigated period these countries have split into several independent countries.

In Table S1 we summarize the number of over-expressions observed in the clusters identified by the Infomap in the weighted networks. We also report in parenthesis the number of distinct clusters where at least one over-expression has been observed. By comparing the number of over-expressions with the number of characterized clusters, one can estimate the average number of over-expressions per cluster. This number decreases, for any considered classification, when we move from the weighted adjacency network to the weighted FDR network and then to the weighted Bonferroni network (the only exception being observed for the language characterization when moving from the FDR to the Bonferroni network). For example, in the case of the genre over-expression, the average number of over-expressions per cluster is 1.41, 1.34 and 1.33 for the adjacency, FDR and Bonferroni network, respectively. The decrease is more pronounced for the genre and filming location characterization. This observation quantitatively indicates a higher specificity in cluster characterization for the statistically validated networks. In the next section, we comment in detail two specific cases, in order to illustrate some of the changes of sensitivity and specificity in the over-expression characterization of clusters in the different weighted networks.

Case studies

We discuss the case of the largest cluster (cluster 1) observed in the partition of the adjacency weighted network. This is a cluster of 6,523 movies mainly in English and mainly produced in the USA. More precisely, the over-expressions that characterize this cluster are: Production country - USA (6,344 movies); Language - English (6,264) and Vietnamese (17); Genre - Comedy (2,385), Thriller (1,300), Action (1,001), Romance (707), Crime (666), Horror (491), Family (441) Adventure (425), Sci-Fi (394), Fantasy (313), Animation (293), Mystery (284), Sport (125) and Western (70); Filming location - Los Angeles, CA (2459), San Francisco, CA (159), Pasadena, CA (141), Santa Clarita, CA (136), Las vegas, NV (135), Long Beach, CA (129), Culver City, CA (121), Burbank, CA (115), California (103), Santa Monica, CA (84) and other 87 locations. The over-expressed production country is USA and in fact 6,344 movies of the cluster have been produced or co-produced in that country. The over-expressed languages are English and Vietnamese. However, it should be noted that the number of movies filmed in these languages is quite different. In fact there are 6,264 movies where the language is English and only 17 movies where the language is Vietnamese. The over-expression of Vietnamese is observed because only 64 movies in

Vietnamese are present in the weighted adjacency network. The genre over-expression involves 14 different genres and the filming location over-expression involves 97 different locations. The large majority of filming locations are in California but cities of many other states are also observed.

We observe that 3,600 movies of the above described adjacency network cluster are split in many clusters of the weighted FDR network. In Table S2 we report some information about the seven FDR network clusters having the largest intersection with the considered adjacency network cluster. Cluster labels in the Table are those provided by the Infomap. The complete list of clusters and movies for all the networks is available upon request to the authors. From the Table it is evident that the size of the FDR clusters is more than one order of magnitude smaller than the size of the adjacency cluster. In other words the Infomap partitioning of the FDR network is quite refined for USA movies. These clusters of movies are almost always characterized by USA as production country and English as language. The genre and filming location over-expression provide the main characterization of FDR clusters. Table S2 shows that the FDR cluster 17 is mainly a cluster of animation movies and, of course, for this cluster no filming location over-expression is observed except the indication of not available (NA). Cluster 56 is a cluster of action movies and the main filming location of them is Los Angeles, CA. Clusters 47, 91 and 123 are all clusters of comedy movies and the over-expressed filming location is again Los Angeles, CA. Cluster 188 is composed by a group of horror movies and a group of science fiction movies, while cluster 95 mainly includes thriller and crime movies. Interestingly, for this last cluster the over-expressed filming locations are all in Florida. This case study shows that the FDR network loses in sensitivity with respect to the adjacency network (less movies are involved in the FDR network) but significantly gains in specificity (clusters in the FDR network are more homogeneous, especially with respect to the genre and filming location characterization). To provide a further example of this improvement in specificity it is worth noting that 8 of the 17 movies in Vietnamese language present in the largest cluster of the weighted adjacency network are found in cluster 775 of the FDR network, which is composed by only 15 movies.

The second case study concerns a cluster of Indian movies. The weighted adjacency movie network presents five large clusters of Indian movies. Here we discuss the properties of the second largest cluster of these five Indian movies clusters. We do not consider the largest one, because it is already very homogeneous according to the language: 90% of indian movies in this cluster are in Hindi. We indicate the selected cluster of Indian movies as cluster 24 of the weighted adjacency network. This cluster consists

of 648 movies, and the over-expressions that characterize this cluster are: Production country - India (643 movies); Language - Telugu (438), Tamil (196), Hindi (52) and Kannada (14); Genre - Drama (312), Action (213), Romance (180), Family (53) and Musical (48); Filming location - Not Available (515), Hyderabad (53), Chennai (31), India (17), Andhra Pradesh (8), Rajahmundry (5), Tamil Nadu (4) and Vikarabad (3). The over-expressed production country is indeed India and over-expressions are also observed for four languages spoken in India, five distinct movie genres, and eight filming locations. By comparing the clusters of the weighted FDR network with this cluster of the weighted adjacency network, we observe a large overlapping of movies. For example, 515 movies of cluster 24 are present in clusters 5 and 43 of the weighted FDR network. In other words, the Indian cluster 24 detected in the weighted adjacency network splits into two distinct clusters in the weighted FDR network. The first cluster (cluster 5) comprises movies where the language spoken is mainly Telugu, whereas the second cluster (cluster 43) mainly comprises movies in Tamil. The characterization of genre and filming location is more specific than the one observed for cluster 24 of the adjacency network, but the degree of specificity is not too high (see the first two columns of Table S3).

A higher degree of specificity is observed when we consider the clusters of the weighted Bonferroni network. In Table S3, we show the over-expression characterization of the five largest clusters of Bonferroni network overlapping with cluster 24 of the weighted adjacency network (last five columns of Table S3). There is a unique language characterization per cluster at this level. The filming location characterization is poor due to the fact that this information is often absent for Indian movies recorded in the IMDb. In fact the “not available” (NA) over-expression is the most frequent one.

In summary, we also notice for Indian movies the ability of statistically validated networks to describe communities of movies that are smaller but more homogeneous, according to the considered classifications, with respect to the communities of movies in the adjacency network.

References

1. Girvan M, Newman MEJ (2002) Community structure in social and biological networks. Proc Natl Acad Sci USA 99: 7821-7826.

2. Fortunato S (2010) Community detection in graphs. Physics Reports 486: 75-174.

3. Rosvall M, Bergstrom CT (2008) Maps of random walks on complex networks reveal community structure. Proc Natl Acad Sci USA 105: 1118-1123.

4. Newman MEJ (2001) Scientific collaboration networks. ii. shortest paths, weighted networks, and centrality. Phys Rev E 64: 016132.

5. Danon L, Diaz-Guilera A, Duch J, Arenas A (2005) Comparing community structure identification. J Stat Mech-Theory Exp : P09008.

6. Tumminello M, Micciché S, Lillo F, Varho J, Piilo J, et al. (2011) Community characterization of heterogeneous complex systems. J Stat Mech-Theory Exp : P01019.
